# Norms According to Age and Gender for the Spanish Version of the Inventory of Depression and Anxiety Symptoms (IDAS-II)

**DOI:** 10.3389/fpsyg.2021.748025

**Published:** 2021-10-08

**Authors:** Manuel Sanchez-Garcia, Ana De la Rosa-Cáceres, Sara Stasik-O'Brien, Juan José Mancheño-Barba, Óscar M. Lozano, Carmen Diaz-Batanero

**Affiliations:** ^1^Department of Clinical and Experimental Psychology, University of Huelva, Huelva, Spain; ^2^Department of Psychology, Knox College, Galesburg, IL, United States; ^3^Community Mental Health Unit, Hospital Juan Ramón Jiménez, Huelva, Spain

**Keywords:** IDAS-II, norms, emotional disorders, unidimensionality, gender

## Abstract

Inventory of Depression and Anxiety Symptoms-II (IDAS-II) constitutes a useful measurement tool with demonstrated psychometric properties that is contributing to the advancement of knowledge of emotional disorders within transdiagnostic models. To implement its use in clinical settings it is important that the scores can be interpreted in order to guide clinical decisions. This study aims to develops normative data for the Spanish version of the IDAS-II. An anonymous online survey was applied to 1,072 subjects, recruited through a stratified random sampling procedure taking into account population gender, age, and geographical region of Spain. Results show that women tend to score higher than men, particularly on the Dysphoria, General Depression, Appetite Gain, and Lassitude scales. Largest effect sizes for differences in the scores according to age were found for Lassitude, Dysphoria, and General Depression. Therefore, normative data according to gender and age group for each IDAS-II scale is provided. The norms provided in this work complement those already available, facilitating the decision-making of clinical professionals. Evidence of unidimensionality is provided for the 19 IDAS-II scales that allows researchers and clinicians to use specific IDAS-II scales independently.

## Introduction

Emotional disorders such as depression and anxiety constitute some of the most prevalent psychopathological conditions (World Health Organization., 2017). These disorders are more disabling and result in a poorer quality of life when compared with other mental disorders such as alcohol use disorder or somatoform disorders and chronic diseases such as diabetes, arthritis, or asthma (Alonso et al., 2004; Moussavi et al., 2007; Grandes et al., 2011). In addition, diagnostic comorbidity, with rates reaching 40–60% of patients (Ruscio et al., 2017; Cancino et al., 2018), accentuates the severity of the disorder (Kessler et al., 2015), and the decline in quality of life (Rapaport et al., 2005).

In the last decade, treatment of this comorbidity has been facilitated by transdiagnostic interventions [e.g., the Unified Protocol–(UP), (Barlow et al., 2011)]. These have been shown to be effective in simultaneously treating several emotional disorders (Barlow et al., 2017; Steele et al., 2018). For example, a recent systematic review revealed that interventions such as the UP (Sakiris and Berle, 2019) are highly effective in reducing the severity of various emotional disorders [e.g., anxiety, depression, general anxiety disorder (GAD), obsessive-compulsive disorder (OCD), panic disorder (PD), and social anxiety disorder], indicating the greater efficiency of this approach in comparison with disorder-specific interventions (Barlow et al., 2017).

From a measurement perspective, transdiagnostic interventions use, on average, more than three instruments to evaluate various disorders (Sakiris and Berle, 2019). As a result, assessment of the impact of interventions is dependent upon the ability of clinicians to interpret the various different scores, since these instruments generally differ in their scoring procedures, assessment timeframes, and measurement scales. In addition, authors such as Fried and Nesse (2015) have pointed out that some of the most widely used assessment instruments [e.g., *Beck Depression Inventory* (BDI; Beck et al., 1996) or the *Hamilton Rating Scale for Depression* (HRSD; Hamilton, 1960)] provide only an overall score of disorder severity. This overall score is generated from summing each of the symptom scores, based on the assumption that all symptoms are interchangeable indicators of the construct (Bollen, 1989; Fried and Nesse, 2015). Consequently, this mode of scoring fails to capture the heterogeneity among people with the same diagnosis, since people with the same overall score may not share common symptoms (Olbert et al., 2014; Fried and Nesse, 2015).

One instrument that, to a large extent, overcomes these limitations is the Inventory of Depression and Anxiety Symptoms-II (IDAS-II) (Watson et al., 2012). This instrument was designed to assess both depression and anxiety symptoms (in order to address the extensive comorbidity between these two types of conditions) as well as to provide comprehensive and dimensional measurement of specific depression and anxiety symptoms. The original version of the instrument (IDAS; Watson et al., 2007) evaluates symptoms of depression and anxiety using 11 non-overlapping scales (Dysphoria, Panic, Social Anxiety, Insomnia, Lassitude, Ill Temper, Traumatic Intrusions, Well-Being, Suicidality, Appetite Loss, and Appetite Gain). In addition, it includes a scale that yields an overall depression score (General Depression), which contains items overlapping with several other IDAS scales. The IDAS-II expanded the instrument's coverage by adding seven additional scales (Traumatic Avoidance, Checking, Ordering, Cleaning, Claustrophobia, Mania, and Euphoria), thus providing dimensional scores for the severity of a wide range of symptoms associated with the following emotional disorders: major depression (MD), GAD, bipolar disorder (BD), posttraumatic stress disorder (PTSD), PD, social phobia, specific phobia, and OCD (Watson et al., 2008, 2012). Thus, the symptoms that constitute each disorder are evaluated with different items grouped into scales. This allows for testing their psychometric properties against other instruments that use a single item for the evaluation of each symptom. In addition, from a clinical perspective, this allows the identification of psychopathological profiles based on the symptoms, which helps clinicians to plan symptom-oriented interventions, whilst also making it possible to evaluate their impact (Kotov et al., 2017; Nelson et al., 2018; Bullis et al., 2019).

A further notable feature of the IDAS-II is that it provides a dimensional score. This increases its sensitivity, particularly for the detection of heterogeneity among patients, while also allowing for the evaluation of clinical change following interventions (Kraemer et al., 2004). Despite these advantages, it should be noted that for the dimensional assessment of symptoms to be useful in clinical practice, it is important that the scores can be interpreted in order to guide diagnosis and decision-making with regard to treatment, choice of drugs, or hospitalization (Kraemer et al., 2004; Widiger and Samuel, 2005).

Currently, normative data are available for the 12 IDAS scales (Stasik-O'Brien et al., 2018), and the 19 scales of the IDAS-II instrument (Nelson et al., 2018). However, these are, to a certain extent, limited with regard to their applicability. First, the IDAS only provides scores associated with three percentiles (70, 94, and 99) (Stasik-O'Brien et al., 2018). In addition, and as the authors point out (Stasik-O'Brien et al., 2018), the development of these norms was based on data from samples that include patients, which affects the level of severity of the scores provided. Further, the two studies mentioned only provide normative data for the English version of the instrument, and no such data are currently available for the Spanish adaptation (De la Rosa-Cáceres et al., 2020). Finally, although there is a growing body of literature to indicate the existence of gender and age differences in emotional disturbances, the available instruments only provide scores for the general population. Research indicates that women score higher on scales used to assess depression and anxiety (Klose and Jacobi, 2004; Kessler et al., 2015; Nelson et al., 2018), whilst older people show a more marked decrease in emotional disturbance symptoms (Hoertel et al., 2015; Patten et al., 2016; Nelson et al., 2018). The influence of gender and age on emotional disorder symptom scores (e.g., Bromet et al., 2011; Patten et al., 2016) highlights the need to provide population-based norms that could be used to highlight differences according to these demographic variables.

Therefore, the primary objective of this study is to develop normative data for the Spanish version of the IDAS-II, obtained from a community sample. Second, we aim to analyze the relationship between the gender and age variables and the IDAS-II scale scores considering both the statistical significance and effect size. If, as suggested by the literature, it is found that scale scores differ significantly as a function of gender and age, then gender- and age-differentiated norms will be constructed for each IDAS-II scale.

## Methods

### Sample

The study was conducted through an anonymous online survey carried out with the specialized company Netquest. This company has more than 155,000 members who were selected via random sampling in the Spanish population. The sample used on present study, composed of 1,072 subjects, was recruited from these panelists through a stratified random sampling procedure (margin of error = 3%, 95% confidence level, distribution p = q), divided into strata representative of the Spanish population for gender, age (ranging between 18 and 75 years) and geographical region of Spain. Fifty percent of this sample were women, and the mean age of participants was 44.32 years (SD = 14.68). Of the sample, 60% had a partner, while 30% were single. In terms of employment status, 62% of the respondents reported being in paid employment. Regarding educational status, 46% had completed higher education (doctorate, master's, or university graduate), and 40% had completed high school and vocational training. Relating geographical representativeness, the distribution of sample is composed as follows: Northwest (Galicia, Asturias) 9%; North center (Vasque country, Cantabria) 9%; North east (Aragon, Catalonia, La Rioja) 12%; Center (Castilla La Mancha, Castilla León, Madrid, Extremadura) 10%; East (Valencia, Murcia, Balearic islands) 15%; South (Andalusia) 19%; metropolitan area of Madrid 12%; metropolitan area of Barcelona 9%; and Canary islands, Ceuta and Melilla 5%

### Instrument

Inventory of Depression and Anxiety Symptoms-II (Watson et al., 2012; Spanish version, De la Rosa-Cáceres et al., 2020). The IDAS-II consists of 99 items rated on a Likert response scale (1 = *not at all* to 5 = *extremely*). Each of the items refers to the presence, during the last 2 weeks, of symptoms of emotional disorders that are grouped into higher hierarchical structures following the HiTOP model (Kotov et al., 2017; De la Rosa-Cáceres et al., 2020). The 99 items are grouped on 18 non-overlapping scales: Dysphoria (e.g., “I felt discouraged about things”), Lassitude (e.g., “I felt exhausted”), Insomnia (e.g., “I had trouble falling asleep”), Suicidality (e.g., “I hurt myself purposely”), Appetite Loss (e.g., “I did not have much of an appetite”), Appetite Gain (e.g., “I thought a lot about food”), Well-Being (e.g., “I felt that I had accomplished a lot”), Ill-Temper (e.g., “I felt like breaking things”), Mania (e.g., “I kept racing from one activity to the next”), Euphoria (e.g., “I felt elated for no reason”), Panic (e.g., “I felt faint”), social anxiety (e.g., “I was anxious about talking in public”), Claustrophobia (e.g., “I avoided tight, enclosed spaces”), Traumatic Intrusions (e.g., “I had memories of something scary that happened”), Traumatic Avoidance (e.g., “I avoided situations that bring up bad memories”), Checking (e.g., “I checked things over and over again”), ordering (e.g., “I felt compelled to follow certain rules”), and Cleaning (e.g., “I avoided handling dirty things”). A General Depression overlapping scale is composed of the 10 Dysphoria items plus two items from each of the following scales: Suicidality, Lassitude, Insomnia, Appetite Loss, and Well-Being (these items are reverse-keyed).

### Procedure

The field work was carried out by a specialized company (Netquest) with panels certified with the ISO 26362 standard. The sample for present study was recruited through a stratified random sampling procedure (considering age, gender, and geographical representativeness) extracted out of the 155,000 panelist of the company. The participation rate was 96.84% of the panelists invited to participate. Sociodemographic equivalence with respect non-respondents was ensured, inviting participants who meet the target characteristics in case another participant decline to participate.

The IDAS-II was administered online (this instrument can be self-administered, as established by the authors of the original version; Watson et al., 2012). Each person selected at random received a pre-test of their reading and comprehension abilities, to check that no automatic responses are made. Once it had been determined that the person was fit to complete the test, the test was administered. Participants were rewarded for their participation. This study was approved by the Ethics Committee of Research Centers in the province of Huelva (Junta de Andalucía, Spain) (file number PI 040/18).

### Statistical Analysis

The normality of the global scores of the 19 IDAS-II scales was checked using Lilliefors correction based on the Kolmogorov-Smirnov procedure. None of the IDAS-II scales follow a normal distribution, particularly the *Suicidality* scores, with values of asymmetry (3.65) and kurtosis (16.48) that are very far from normal.

Before generating the standardized scores, the unidimensionality of the IDAS-II scales was determined. For this purpose, various indices of unidimensionality were calculated: (a) percentage of variance explained by the first factor, considering that the scale is one-dimensional if the first factor explains at least 40% of the variance of all items (Carmines and Zeller, 1979); (b) the number of dimensions determined by the minimum average partial (MAP) test (Velicer, 1976; Velicer et al., 2000); (c) Horn's parallel analysis (PA) method (Horn, 1965); (d) Ruscio and Roche's comparison data (CD) technique (Ruscio and Roche, 2012); (e) confirmatory factor analysis fit indices for a one-dimensional model such as the Root Mean-Square Error of Approximation (RMSEA), the Root Mean Square Residual (RMSR), the Tucker Lewis Index (TLI) and Comparative Fit Index (CFI). Values below 0.08 for RMSEA and RMSR and above 0.90 for the CFI and TLI are taken to indicate a good fit (Hu and Bentler, 1999).

Having demonstrated sufficient evidence of unidimensionality of the IDAS-II scales, standardized percentiles and *T*-scores were generated for each scale for the whole sample. Various tests were then conducted to compare means (*t*-tests and ANOVAs) to confirm whether there were differences according to gender and age. In all these tests, statistical significance and the effect size were both taken into account. According to Cohen's guidelines (Cohen, 1988, 1992) *d*-values from |0.20| to |0.49| are taken to indicate small effect sizes; those ranging from |0.50| to |0.79| are considered medium; and values above |0.80| are considered large effect sizes. Eta-squared values from |0.01| to |0.05| represent small effect sizes; those ranging from |0.06| to |0.13| indicate medium effect sizes; and values above |0.14| indicate large effect sizes (Cohen, 1988, 1992). Differences according to gender and age were subsequently determined for each of the IDAS-II scales.

Descriptive statistics, internal consistency (alpha and omega), exploratory factor analysis, and tests for the comparison of means were conducted with SPSS version 25. For the dimensionality statistics, the functions “vss” and “fa.parallel” of the R package “psych” (Revelle, 2019); the function “cfa” of the “lavaan” R package (Rosseel, 2012); and the function “CD” of the “EFAtools” R package (Steiner and Grieder, 2020) were used. The “psych” R package was used to calculate the omega reliability coefficients. The percentile values were obtained from the SPSS percentile calculation function.

## Results

### Descriptive Statistics and Reliability Estimates

Table 1 shows the descriptive statistics and internal consistency of the IDAS-II scales. As can be observed, the internal consistency values can be considered adequate, ranging from α = 0.74 (ω = 0.77) and α = 0.91 (ω = 0.93), with a mean α-value of 0.83 (mean ω-value of 0.88).

**Table 1:** Reliability, means, and standard deviations for IDAS-II scales.

**Scale**	**No. of items**	**This study (α)**	**This study (ω)**	**This study Spain—community sample—online (*N* = 1,072)**	**Nelson et al. (2018) USA—community sample—online (*N* = 1,836)**	***p^a^***	***d^a^***	**De la Rosa-Cáceres et al. (2020) Spain—community sample (*N* = 620)**	***p^a^***	***d^a^***
				***M(SD)***	***M(SD)***			***M(SD)***		
General depression	20	0.91	0.92	39.82(12.17)	41.94(14.75)	<0.001	0.15	42.09(21.05)	0.005	0.14
Dysphoria	10	0.90	0.90	19.18(7.44)	19.90(8.61)	0.022	0.09	20.82(11.03)	<0.001	0.18
Lassitude	6	0.79	0.81	11.13(4.25)	13.14(5.23)	<0.001	0.41	12.66(6.80)	<0.001	0.29
Insomnia	6	0.89	0.93	12.02(5.37)	12.43(5.54)	0.052	0.07	13.31(7.46)	<0.001	0.21
Suicidality	6	0.84	0.91	7.27(2.86)	8.35(3.93)	<0.001	0.30	7.27(3.66)	0.999	0.00
Appetite loss	3	0.84	0.88	4.57(2.19)	5.06(2.82)	<0.001	0.19	4.63(2.64)	0.615	0.03
Appetite gain	3	0.80	0.83	5.59(2.69)	6.19(2.87)	<0.001	0.21	6.01(3.49)	0.006	0.14
Well-being	8	0.84	0.88	22.41(5.83)	22.40(7.42)	0.970	0.01	23.56(8.97)	0.001	0.16
Ill-temper	5	0.86	0.91	8.91(3.98)	8.15(4.01)	<0.001	0.19	9.90(5.63)	<0.001	0.21
Mania	5	0.80	0.86	8.46(3.69)	9.24(4.34)	<0.001	0.19	10.26(5.79)	<0.001	0.39
Euphoria	5	0.77	0.86	7.87(3.20)	8.84(3.55)	<0.001	0.28	9.08(5.11)	<0.001	0.30
Panic	8	0.89	0.92	11.53(4.92)	11.92(5.69)	0.061	0.07	13.23(7.91)	<0.001	0.27
Social anxiety	6	0.82	0.88	9.44(4.11)	10.86(5.51)	<0.001	0.28	10.53(6.36)	<0.001	0.22
Claustrophobia	5	0.85	0.91	7.35(3.66)	7.25(3.70)	0.480	0.03	7.84(5.00)	0.021	0.11
Truamtic intrusions	4	0.86	0.92	6.02(2.99)	6.60(3.50)	<0.001	0.17	6.71(4.18)	<0.001	0.20
Traumatic avoidance	4	0.83	0.90	7.76(3.70)	7.85(3.86)	0.538	0.02	8.54(4.73)	<0.001	0.19
Checking	3	0.78	0.83	5.19(2.44)	6.18(2.86)	<0.001	0.36	6.02(3.18)	<0.001	0.30
Ordering	5	0.74	0.77	9.70(3.69)	8.78(3.93)	<0.001	0.24	10.15(5.47)	0.044	0.10
Cleaning	7	0.84	0.88	12.42(4.50)	11.83(5.05)	0.002	0.12	11.59(7.31)	0.004	0.15

*d, absolute value of Cohen's d; α, internal consistency alpha; ω, internal consistency omega1*.

a *Comparison with present sample*.

Comparison of the mean scores with those reported by Nelson et al. (2018) for the U.S. sample and the sample of De la Rosa-Cáceres et al. (2020) revealed that the differences are statistically significant for almost all of the scales. However, statistical significance is driven more by sample size than effect size, since 12 of the 19 comparisons with the Nelson et al. study yielded effect size values (d) below 0.20. Compared with the data from De la Rosa-Cáceres et al. (2020), 11 subscales have effect size values below 0.20 (Nelson et al., 2018). The highest effect size value corresponds to the difference in mean scores for Mania (*d* = 0.39).

### Unidimensionality of the Scales

The 18 IDAS-II scales provide evidence of unidimensionality in their scores for most analysis carried out. Although CD analysis offers a higher number of factors on most scales, MAP and PA analysis suggested a unidimensional solution to be the best option for all specific scales. The percentages of variance explained by the first factor range from 47% (Well-Being, 8 items) to 76% (Appetite Loss, 3 items) and the RMSR and TLI-values indicate an adequate one-dimensional model fit for all of the scales except *Suicidality* (RMSEA = 0.23; RMSR = 0.10; CFI = 0.84; TLI = 0.73) and *Ordering* (RMSEA = 0.26; RMSR = 0.11; CFI = 0.75; TLI = 0.49). However, in both cases, the values of the fit indices increase significantly if we estimate the correlation between the measurement errors of items with highly overlapping contents: in the Suicidality scale, Items 22 and 38 refer to self-directed harmful actions or behaviors, while the rest of the items refer to thoughts (RMSEA = 0.14; RMSR = 0.05; CFI = 0.94; TLI = 0.89). In the Ordering scale, items 65 and 69 refer to reorganization (action and need to reorganize) whilst the remaining items refer to habits and rituals (RMSEA = 0.09; RMSR = 0.04; CFI = 0.98; TLI = 0.94).

The lowest values correspond to the General Depression composite scale (RMSEA = 0.11; RMSR = 0.08; CFI = 0.79; TLI = 0.76). By including the covariance between measurement errors of some of its items [for example, the items of Appetite Loss (items 1 and 26), Dysphoria (items 21 and 31 and items 40 and 48), Suicidality (items 13 and 52), Well-being (items 27 and 64), or Insomnia (items 11 and 51) scales] the values of the fit indices improve significantly (RMSEA = 0.07; RMSR = 0.05; CFI = 0.91; TLI = 0.90).

### Scales General Percentiles

Table 2 shows the percentiles and normalized *T*-scores (standardized from the percentiles) associated with the direct scores of each of the IDAS-II scales. To facilitate the reading and interpretation of the scores, for each of the symptoms, scores are presented for the values corresponding to the mean in *T*-scores (*T* = 50, *P*_50_) and the mean plus/minus one and two standard deviations (*T*-values of 60–40 and 70–30, respectively), which correspond to the P_85_, P_15_, P_97.75_, and P_2.25_.

**Table 2:** Percentiles, T scores, and direct scores for all sample (*N* = 1,072).

***Percentiles***	**General depression**	**Dysphoria**	**Lassitude**	**Insomnia**	**Suicidality**	**Appetite loss**	**Appetite gain**	**Well-being**	**Ill-temper**	**Mania**	**Euphoria**	**Panic**	**Social anxiety**	**Claustrophobia**	**Truamtic intrusions**	**Traumatic avoidance**	**Checking**	**Ordering**	**Cleaning**	***Normalized T scores***
***1***	23							10												***27***
***2.25***	24							11												***30***
***5***	25	10	6					12										5	7	***34***
***10***	26	11		6				14	5											***37***
***15***	28	12	7				3	16		5	5	8	6			4	3		8	***40***
***20***	29			7														6		***42***
***25***	31	13	8			3		18						5	4				9	***43***
***30***	32	14		8	6			19	6									7		***45***
***35***	33	15	9	9			4	20		6			7			5			10	***46***
***40***	34	16						21	7		6	9				6	4	8		***47***
***45***	36	17		10				22		7									11	***49***
***50***	37	18	10	11			5		8		7		8			7		9		***50***
***55***	39		11					23		8		10		6	5				12	***51***
***60***	40	19		12		4		24	9				9			8	5	10	13	***53***
***65***	42	20	12	13		5	6	25		9	8	11	10	7	6			11		***54***
***70***	44	22		14	7			26	10			12		8		9	6		14	***55***
***75***	47	23	13	15		6	7	27	11	10	9	13	11	9	7	10		12	15	***57***
***80***	49	25	14	16			8	28	12	11	10	14	12	10	8	11	7	13	16	***58***
***85***	53	27	15	18	8	7		29	13	12	11	16	14	11	9	12	8	14	17	***60***
***90***	57	30	17	20	10	8	9	30	15	14	13	19	16	13	10	13	9	15	19	***63***
***95***	63	34	20	23	14	9	11		17	16	15	23	18	15	12	15	10	16	22	***66***
***97***	68	36	21	25	16	10	12	32	19		16	25	20	17	14	16	11		23	***69***
***97.75***	70	38	22		17	11	13	33		18	17	26	21		15	17	12	18	24	***70***
***99***	79	44	24	28	19	12	15	35	22	20	18	30	23	20	17	18	13	20	25	***73***

*Underlined: P_2.25_, P_15_, P_50_, P_85_, P_97.75_*.

### Group Comparisons

Table 3 shows the comparisons of the mean IDAS-II scale scores according to gender and age group. Statistically significant gender differences are found with small and medium effect sizes for all scales except Suicidality, Well-Being, Euphoria, and Ordering. The largest effect sizes correspond to the comparisons for Dysphoria (*d* = 0.50), General Depression (*d* = 0.49), Appetite Gain (*d* = 0.49), and Lassitude (*d* = 0.44). When looking at the differences between age groups, statistically significant differences are observed with small or medium effect sizes for all scales except well-being, with the largest effect sizes being found for Lassitude (η^2^ = 0.12), Dysphoria (η^2^ = 0.10), and General Depression (η^2^ = 0.08).

**Table 3:** Mean (Standard Deviation) by gender and age group.

**Scale**	**Men (*n* = 537)**	**Women (*n* = 535)**	***p***	***d***	**18–29 years (*n* = 223)**	**30–44 years (*n* = 344)**	**45–54 years (*n* = 202)**	**55–75 years (*n* = 303)**	***p***	**η^2^**
	***M(SD)***	***M(SD)***			***M(SD)***	***M(SD)***	***M(SD)***	***M(SD)***		
General depression	36.93(10.87)	42.72(12.70)	<0.001	0.49	45.17(13.21)	40.74(11.86)	39.06(12.14)	35.35(9.79)	<0.001	0.08
Dysphoria	17.38(6.48)	20.99(7.89)	<0.001	0.50	22.82(8.27)	19.72(7.24)	18.61(7.20)	16.28(5.76)	<0.001	0.10
Lassitude	10.21(3.92)	12.05(4.36)	<0.001	0.44	13.42(4.85)	11.60(4.10)	10.57(3.83)	9.27(3.13)	<0.001	0.12
Insomnia	11.34(4.95)	12.70(5.68)	<0.001	0.26	12.59(5.46)	12.40(5.61)	12.04(5.36)	11.15(4.93)	0.007	0.01
Suicidality	7.19(2.76)	7.35(2.95)	0.356	0.06	8.17(3.90)	7.06(2.51)	7.16(2.90)	6.91(2.04)	<0.001	0.03
Appetite loss	4.31(2.05)	4.83(2.28)	<0.001	0.24	5.18(2.37)	4.58(2.13)	4.40(2.18)	4.22(2.00)	<0.001	0.03
Appetite gain	4.96(2.21)	6.23(2.96)	<0.001	0.49	7.14(3.10)	5.71(2.76)	5.19(2.36)	4.59(1.82)	<0.001	0.11
Well-being	22.55(5.67)	22.26(5.97)	0.404	0.05	21.86(6.08)	22.60(5.87)	22.10(5.75)	22.79(5.62)	0.238	0.01
Ill-temper	8.18(3.52)	9.64(4.26)	<0.001	0.37	10.46(4.75)	9.31(3.94)	8.49(3.84)	7.60(2.84)	<0.001	0.07
Mania	7.81(3.26)	9.10(3.96)	<0.001	0.36	9.87(4.26)	8.76(3.85)	8.06(3.34)	7.33(2.75)	<0.001	0.06
Euphoria	7.78(3.20)	7.95(3.19)	0.382	0.05	8.93(3.79)	7.80(3.13)	7.35(2.63)	7.50(2.96)	<0.001	0.03
Panic	10.75(4.35)	12.32(5.31)	<0.001	0.32	13.14(5.74)	11.76(5.16)	11.09(4.62)	10.37(3.66)	<0.001	0.04
Social anxiety	8.96(3.78)	9.93(4.36)	<0.001	0.24	11.38(5.18)	9.40(3.79)	8.93(3.66)	8.42(3.27)	<0.001	0.07
Claustrophobia	6.95(3.33)	7.75(3.92)	<0.001	0.22	8.09(4.25)	7.22(3.49)	6.92(3.02)	7.24(3.70)	0.006	0.01
Truamtic intrusions	5.62(2.61)	6.41(3.27)	<0.001	0.27	7.11(3.66)	6.05(2.92)	5.69(2.73)	5.40(2.41)	<0.001	0.04
Traumatic avoidance	7.38(3.59)	8.14(3.77)	0.001	0.21	8.89(4.17)	7.45(3.48)	7.42(3.45)	7.51(3.58)	<0.001	0.03
Checking	4.96(2.14)	5.41(2.68)	0.002	0.19	6.11(3.05)	5.12(2.36)	4.77(1.97)	4.86(2.10)	<0.001	0.04
Ordering	9.51(3.53)	9.90(3.84)	0.082	0.11	10.54(4.30)	9.63(3.67)	9.04(3.22)	9.61(3.41)	<0.001	0.02
Cleaning	11.81(4.00)	13.03(4.87)	<0.001	0.27	13.03(4.74)	12.73(4.65)	12.01(4.21)	11.89(4.24)	0.009	0.01

*d, absolute value of Cohen's d; η^2^, eta-squared*.

### Percentile Scores According to Age and Gender

Given the differences found, Tables 4–7 present the scores obtained as a function of gender and age variables, according to the following participant categories (in order not to generate subgroups with reduced sample sizes, they will be grouped into two age groups): (1) men aged 18–44 years (*n* = 188) (Table 4); (2) men aged 45–75 years (*n* = 349) (Table 5); (3) women aged 18–44 years (*n* = 379) (Table 6); and (4) women aged 45–75 years (*n* = 156) (Table 7).

**Table 4:** Percentiles, T scores, and direct scores for 18–44 years old men (*n* = 188).

***Percentiles***	**General depression**	**Dysphoria**	**Lassitude**	**Insomnia**	**Suicidality**	**Appetite loss**	**Appetite gain**	**Well-being**	**Ill-temper**	**Mania**	**Euphoria**	**Panic**	**Social anxiety**	**Claustrophobia**	**Truamtic intrusions**	**Traumatic avoidance**	**Checking**	**Ordering**	**Cleaning**	***Normalized T scores***
***1***	24							10												***27***
***2.25***	25	10	6	6				11											7	***30***
***5***	26	11						13	5									5		***34***
***10***	27		7				3	15		5	5		6			4				***37***
***15***	28	12		7				16				8			4		3		8	***40***
***20***	30	13	8			3		18										6		***42***
***25***	31	14	9	8	6			19	6					5					9	***43***
***30***	33	15		9						6			7					7		***45***
***35***	34	16	10				4	20	7		6					5	4	8	10	***46***
***40***	36	17						21		7		9	8			6				***47***
***45***	37			10															11	***49***
***50***	38	18	11	11			5	22	8	8	7	10	9		5	7	5	9	12	***50***
***55***	39	19				4		23			8							10		***51***
***60***	40	20	12	12			6	24	9	9		11	10	6		8			13	***53***
***65***	42	21	13	13		5		25			9		11	7	6		6	11	14	***54***
***70***	44	22	14	14	7		7		10	10	10	12		8		9		12		***55***
***75***	47	24				6		26	11	11		13	12	9	7	10	7	13	15	***57***
***80***	49	26	15	15	8		8	27	12	12	11	15	14	10	8	11		14	16	***58***
***85***	55	28	16	18	9	7	9	28	14	14	13	16	15	12	10	13	8	15	17	***60***
***90***	59	30	18	20	13	9		30	15	15	14	20	17	14	11		9		18	***63***
***95***	63	34		23	17		10	32	17	17	16	24	21	16		16	10	17	21	***66***
***97***	67	35	21		18		13				17	27	22		13		11	18	23	***69***
***97.75***	68	36	22	24	19	10		33	19		18	28		17	14	17	12		24	***70***
***99***	71	39	25	26	21	12	14	34	22	18	20	30	23	19	19	18	13	19	25	***73***

*Underlined: P_2.25_, P_15_, P_50_, P_85_, P_97.75_*.

**Table 5:** Percentiles, T scores, and direct scores for 45–75 years old men (*n* = 349).

***Percentiles***	**General depression**	**Dysphoria**	**Lassitude**	**Insomnia**	**Suicidality**	**Appetite loss**	**Appetite gain**	**Well-being**	**Ill-temper**	**Mania**	**Euphoria**	**Panic**	**Social anxiety**	**Claustrophobia**	**Truamtic intrusions**	**Traumatic avoidance**	**Checking**	**Ordering**	**Cleaning**	***Normalized T scores***
***1***	22							9												***27***
***2.25***	23	10						10												***30***
***5***	24							12										5	7	***34***
***10***	25		6	6				15			5									***37***
***15***	26	11					3	16	5	5			6			4	3	6		***40***
***20***								18				8							8	***42***
***25***	28	12		7				19							4					***43***
***30***	29		7		6	3								5				7	9	***45***
***35***	30	13		8				20												***46***
***40***	31		8	9				21	6		6					5		8	10	***47***
***45***		14						22		6						6	4			***49***
***50***	32	15		10			4	23					7					9	11	***50***
***55***	34		9	11				24	7	7	7	9				7				***51***
***60***	35	16																10	12	***53***
***65***	36	17	10	12		4	5	25	8		8	10	8	6	5	8	5			***54***
***70***	39	18		13	7			26		8				7		9		11	13	***55***
***75***	40	19	11	14		5		27	9	9	9	11	10		6		6		14	***57***
***80***	43	21	12	15			6	28	10			12		8		10		12		***58***
***85***	46	22		16	8	6		29	11	10	10	13	12	9	7	11	7	13	15	***60***
***90***	48	24	14	18		7	7	30		11	12	15	13	11	8	12		14	17	***63***
***95***	56	29	16	21	12	9			15	13	14	19	16	14	11	15	9	16	20	***66***
***97***	57	30	18	23	14		9	32	16	15	15	22	17		12	16	10	17		***69***
***97.75***	61	31		24	15	10						23	18	16	13				21	***70***
***99***	67	35	20	26	18	11	11	34	20	17	18	25	20	19	14	17	12	18	23	***73***

*Underlined: P_2.25_, P_15_, P_50_, P_85_, P_97.75_*.

**Table 6:** Percentiles, *T* scores, and direct scores for 18–44 years old women (*n* = 379).

***Percentiles***	**General depression**	**Dysphoria**	**Lassitude**	**Insomnia**	**Suicidality**	**Appetite loss**	**Appetite gain**	**Well-being**	**Ill-Temper**	**Mania**	**Euphoria**	**Panic**	**Social anxiety**	**Claustrophobia**	**Truamtic intrusions**	**Traumatic avoidance**	**Checking**	**Ordering**	**Cleaning**	***Normalized T Scores***
***1***	23																			***27***
***2.25***	24	10	6	6				10										5	7	***30***
***5***	27	11					3	12	5	5	5		6			4				***34***
***10***	30		7					14				8								***37***
***15***	31	13	8	7		3		16									3	6	8	***40***
***20***	33							17	6	6				5	4					***42***
***25***	34	15	9	8			4	18	7							5		7	9	***43***
***30***	36	17		9	6			19			6	9	7							***45***
***35***	37		10	10				20		7						6		8	10	***46***
***40***	38	18					5		8				8			7	4		11	***47***
***45***	40	19	11	11				21				10						9		***49***
***50***	41	20	12	12		4	6	22	9	8	7		9	6	5				12	***50***
***55***	43	21				5		23		9		11				8	5	10	13	***51***
***60***	45	22	13	13			7	24	10		8	12	10	7	6					***53***
***65***	47	24		14				25	11	10		13	11			9	6	11	14	***54***
***70***	49	25	14	15	7	6	8	26		11	9	14	12	8	7			12	15	***55***
***75***	51	27	15	16				27	12	12		15	13	9	8	10	7	13	16	***57***
***80***	53	28	16	17	8		9	28	14	13	10	16	14	10	9	11	8	14	18	***58***
***85***	57	30	17	19	9	7	10	29	15	14	12	18	15	11	10	13	9	15	19	***60***
***90***	60	33	19	22	11	8	11	30	17	16	13	20	17	13	11	14	10	16	20	***63***
***95***	69	38	21	24	13	9	13	32	19	18	15	24	19	17	14	16	12	17	22	***66***
***97***	77	43	23	27	16	10	14	34	20		17	27	22	18	15	17	13	19	24	***69***
***97.75***	78	44	24	28	18	11	15	35	21	19		28	23	19	17				25	***70***
***99***	85	45	26	29	20	12		37	25	22	19	33	26	24	18	19	14	21	26	***73***

*Underlined: P_2.25_, P_15_, P_50_, P_85_, P_97.75_*.

**Table 7:** Percentiles, *T* scores, and direct scores for 45–75 years old women (*n* = 156).

***Percentiles***	**General depression**	**Dysphoria**	**Lassitude**	**Insomnia**	**Suicidality**	**Appetite loss**	**Appetite gain**	**Well-being**	**Ill-temper**	**Mania**	**Euphoria**	**Panic**	**Social anxiety**	**Claustrophobia**	**Truamtic intrusions**	**Traumatic avoidance**	**Checking**	**Ordering**	**Cleaning**	***Normalized T scores***
***1***	24							10												***27***
***2.25***	25	10	6					11										5	7	***30***
***5***	27	11		6			3	12	5	5						4				***34***
***10***	28							14			5	8	6							***37***
***15***	30	12	7					16						5			3	6	8	***40***
***20***	31	13	8	7				17											9	***42***
***25***	33	14		8		3		18	6						4	5		7		***43***
***30***	34			9	6		4			6									10	***45***
***35***		15	9	10				20	7			9	7			6		8		***46***
***40***	35	16						21		7	6					7			11	***47***
***45***	36	17		11										6			4	9		***49***
***50***	38	18	10	12			5	23	8			10	8			8			12	***50***
***55***	39					4				8	7			7	5			10	13	***51***
***60***	40	19	11	13				24				11	9	8		9	5		14	***53***
***65***	42	20				5	6		9	9	8			9	6			11		***54***
***70***	43	22	12	14	7			26		10		12	10			10	6		15	***55***
***75***	48	24		15		6	7		10		9	13	11	10	7			12		***57***
***80***	50	26	13	17				27	11	11	10	14	12	11		11	7		16	***58***
***85***	53	27	14	19	8	7	8	28	12	12		16	13	13	8	12	8	13	19	***60***
***90***	57	31	16	21	9	8	9	29	13		11	19	16	14	10	13		15	21	***63***
***95***	64	34	18	24	13	9	10		16	15	13	22	18	16	13	15		16	23	***66***
***97***	70	35	20		16	11	12	31	17	17	14	25	19	18	16	17	10		24	***69***
***97.75***	73	37	21	25					18	18	15	26		20				17		***70***
**99**	83	44	24	29	21	13	14	36	22	21	17	28	21	24	17	19	12	21	26	**73**

*Underlined: P_2.25_, P_15_, P_50_, P_85_, P_97.75_*.

## Discussion

The IDAS-II has been highlighted as an efficient instrument for evaluating the severity of the symptoms of a wide variety of emotional disorders. In order to facilitate its application and interpretation, this study provides, for the first time, national norms for gender and age of the Spanish version of the IDAS-II using data from a community sample. The importance of these indicators is justified by the observation that women tend to score higher than men, particularly on the dysphoria, general depression, appetite gain, and lassitude scales. Moreover, there are differences in the scores according to age. These differences justify the need to provide normative data according to gender and age group for each IDAS-II scale. Finally, evidence of unidimensionality is provided for the 19 IDAS-II scales which, together with the high values of internal consistency, ensures that each IDAS-II scale can be used independently.

In accordance with our main objective, we have provided normative data for the Spanish version of the IDAS-II based on a community sample. In agreement with previous studies (Nelson et al., 2018; De la Rosa-Cáceres et al., 2020), it is observed that the variables of the IDAS-II are not normally distributed, and so the percentiles corresponding to the scores on each scale are provided. As several authors point out (Crawford and Garthwaite, 2009; Nelson et al., 2018), percentiles calculated from the actual distribution of scores are most useful and informative when the data do not yield a normal distribution. Thus, for example, regarding the value of the mean and standard deviation of Suicidality (7.27 and 2.86, respectively) we could suppose that, if these values followed a normal distribution, a person with a score of 8 would correspond to the 60th percentile, when in fact this score actually places that person at the 85th percentile. This information helps clinicians to correctly interpret the scores and thus facilitates decision making (Chien and Yao, 2014).

Comparing the scores reported here with those of other similar studies, we found that our scores are lower than those reported for the IDAS used by Stasik-O'Brien et al. (2018), but similar to those obtained with the version of the IDAS-II used by Nelson et al. (2018). Thus, the direct scores of 53 for General Depression and 27 for Dysphoria, which, in the work of Stasik-O'Brien et al. (2018) correspond to the 70th percentile, would be placed at the 83rd percentile in the work of Nelson et al. (2018). Moreover, the scores that Stasik-O'Brien et al. (2018) rank as *Mild* (70th percentile), would appear to be closer to the *Moderate* level (94th percentile) according to our data. These discrepancies could be explained by the different samples used; whilst the data reported by both Nelson et al. (2018) and in the present study were based on community sample scores, Stasik-O'Brien et al. (2018) used a combined sample that included patients. In this regard, Stasik-O'Brien et al. (2018) point out that the inclusion of patients increases the severity of the scores (giving rise to higher scores) and thus they recommend that data are taken from community samples such as the one used in the present study.

When comparing our scores with those reported by Nelson et al. (2018), there are certain similarities, particularly between those that correspond to the highest percentiles. For example, the scores associated with the 97th percentile of the Insomnia (score = 25), Cleaning (score = 23) and Traumatic Avoidance (score = 16) scales are similar in both studies. Further, the direct score corresponding to the 85th percentile—which usually marks the point of statistical “abnormality”—is similar (or broadly similar) across several scales (e.g., Insomnia, Traumatic Intrusions, Claustrophobia, Mania, Social Anxiety, and General Depression). Although there are more discrepancies between the low values, there are also some similarities. For example, a score of 4 corresponds to the 25th percentile of Traumatic Intrusions in both studies, whilst the 25th percentile of Well-Being corresponds to a score of 17 points in the study by Nelson et al. (2018), and a score of 18 points in the present study.

Moreover, and consistent with the literature (e.g., Kessler et al., 2015; Patten et al., 2016; Nelson et al., 2018), our results highlight how gender and age variables are associated with depression and anxiety scores, particularly for the *General Depression, Dysphoria, Lassitude*, and *Appetite Gain* scales. The differences found are unsurprising; women score higher than men (Klose and Jacobi, 2004; Bromet et al., 2011; Kessler et al., 2015), and younger people score higher than people of older ages (Hoertel et al., 2015; Kessler et al., 2015; Patten et al., 2016; Nelson et al., 2018). Considering that General Depression evaluates the central symptoms of depression, and Dysphoria is linked to the central and shared aspects of depression and anxiety (Watson et al., 2007, 2012), it seems that the higher scores obtained by women on these two scales is congruent with those studies that have consistently shown a greater prevalence of depression and anxiety in women (e.g., Bromet et al., 2011; Bandelow and Michaelis, 2015; Kessler et al., 2015; Jalnapurkar et al., 2018). Moreover, and similar to how other fatigue-related disorders (e.g., chronic fatigue syndrome and fibromyalgia) have a higher prevalence in women (Wolfe et al., 2018; Lim et al., 2020), our results also seem to indicate that the link between fatigue/lassitude and depression is more strongly associated with females. In line with our findings, Nelson et al. (2018) also found significantly higher Lassitude scores in women, the latter being one of the most gender-influenced symptoms. In light of these findings, it would be of interest to conduct future studies to clarify the relationship between fatigue and depression in women.

The results reported here indicate the importance of addressing how the variables of gender and age are related to symptoms measured by the IDAS-II. Thus, the present work provides, for the first time, normative data for examining age and gender differences for each IDAS-II scale. These differences are particularly evident when observing the scores obtained by women from 18 to 44 years of age and men from 45 to 75 years of age, which differ significantly from the whole sample. The other two subsamples show percentile scores very close to those obtained by the whole sample. Consistent with the literature where female gender and younger ages are associated with higher scores (e.g., Bromet et al., 2011; Hoertel et al., 2015; Kessler et al., 2015), 18–44 year-old women constitute the group with the highest mean scores whilst 45–75 year-old men show the lowest scores. These differences according to gender and age are shown in the percentiles corresponding to scores of the participant categories. For example, a score of 18 on the *Dysphoria* scale, which corresponds to the 50th percentile (*T* = 50) in the whole sample, corresponds to the 40th percentile (*T* = 47) in the subsample of 18–44 year-old women and the 79th percentile (*T* = 55) in the subsample of 45–75 year-old men.

Finally, the present study provides, for the first time, evidence of unidimensionality for all the IDAS-II scales, extending the unidimensionality analyses carried out by Watson et al. (2007) on the 11 scales of the original IDAS. This evidence of unidimensionality, together with the high values of internal consistency of the scales, supports the possibility of generating and using the score of each IDAS-II scale independently. Although some studies have used certain IDAS-II scales independently (e.g., Bauer et al., 2019; Vidaña et al., 2020), our study is the first to provide the evidence of unidimensionality that is necessary for interpreting the scores of the scales in an independent manner. This allows researchers and clinicians to use specific IDAS-II scales without having to administer the entire instrument. The IDAS-II thus makes it possible to evaluate both specific symptom dimensions and broader internalizing dimension (Stanton et al., 2020). This is of particular interest for transferring transdiagnostic approaches into clinical settings, which, although they are providing empirical evidence of interest in the research field, still require tools to facilitate decision-making by clinical professionals (Contreras et al., 2019).

Although the results reported here are of interest, it is necessary to acknowledge certain limitations of the study. In this regard, it should be noted that the data of the community sample used here were collected through an online procedure. Although this procedure has allowed us to efficiently collect data from participants in all geographical regions of Spain, some authors point out that online data collection could lead to higher psychopathological scores in comparison with data gathered from other community samples using traditional procedures (Arditte et al., 2016). In addition, the use of digital media can create an access gap for certain people. In spite of this, the data reported in this study indicate that our sample does not differ from other Spanish samples whose data were collected through traditional methods (De la Rosa-Cáceres et al., 2020) or from the American normative sample used by Nelson et al. (2018). These authors also gathered the IDAS-II data online and found that the scores did not differ from those of other samples evaluated through traditional procedures. Therefore, we consider that the norms provided in this work complement those already available, facilitating the decision-making of clinical professionals and opening up new possibilities for research on the symptoms of emotional disorders.

## Data Availability Statement

The datasets presented in this study can be found in the article/Supplementary Material.

## Ethics Statement

The studies involving human participants were reviewed and approved by the Ethics Committee of Research Centers in the province of Huelva (Junta de Andalucía, Spain) (file number PI 040/18). The patients/participants provided their written informed consent to participate in this study.

## Author Contributions

CD-B, ÓL, and ADR-C has contributed on the conception and design of study. ADR-C, JM-B, and MS-G has contributed on the acquisition of data. ADR-C, MS-G, and ÓL had participated on the analysis of data. ADR-C, SS-ÓB, and CD-B had participated on the interpretation of data and drafted the manuscript. All authors have revised the manuscript critically for intellectual content and approved the final version of the manuscript submitted.

## Funding

This work was supported by the grant Network-Psyco: Modelización a través de redes empíricas de las conexiones entre facetas y rasgos psicológicos project UHU-1257470 on Programa Operativo FEDER Andalucía 2014–2020, provided by Fondo Europeo de Desarrollo Regional (EU) and Junta de Andalucía (Spain) and by the grant FPU19/00144 provided by the Spanish Ministry of Universities. The funding source had no role in the design of this study and will not have any role during its execution, analyses, interpretation of the data, or decision to submit results. Open Access publication fees are supported by Estrategia de Política de investigación y de Transferencia 2021 University of Huelva.

## Conflict of Interest

The authors declare that the research was conducted in the absence of any commercial or financial relationships that could be construed as a potential conflict of interest.

## Publisher's Note

All claims expressed in this article are solely those of the authors and do not necessarily represent those of their affiliated organizations, or those of the publisher, the editors and the reviewers. Any product that may be evaluated in this article, or claim that may be made by its manufacturer, is not guaranteed or endorsed by the publisher.
